# Adherence to Mediterranean diet and its association with multiple colonic polyps of unknown origin: a case-control study

**DOI:** 10.3389/fnut.2023.1186808

**Published:** 2023-06-22

**Authors:** Gabriela Bujanda-Miguel, Alejandro Martínez-Roca, Anabel García-Heredia, David Guill-Berbegal, Enrique Roche, Rodrigo Jover

**Affiliations:** ^1^Servicio de Medicina Digestiva, Hospital General Universitario Dr. Balmis, Instituto de Investigación Biomédica ISABIAL, Alicante, Spain; ^2^Department of Applied Biology-Nutrition, Institute of Bioengineering, University Miguel Hernández, Elche, Spain; ^3^CIBER Fisiopatología de la Obesidad y Nutrición (CIBEROBN), Instituto de Salud Carlos III (ISCIII), Madrid, Spain; ^4^Departamento de Medicina Clínica, Universidad Miguel Hernández, Alicante, Spain

**Keywords:** adenomatous polyps, colorectal cancer, colorectal polyps, diet, serrated polyps

## Abstract

**Introduction:**

Multiple colonic polyps do not have a genetic origin in most patients, and the cause of this phenotype remains elusive. Environmental factors, such as diet, could be related to this phenotype. Our aim was to investigate the relationship between the adherence to Mediterranean diet and multiple colonic polyps of unknown origin.

**Methods:**

A case-control pilot study was carried out with a sample of 38 individuals, including 23 cases with more than 10 adenomatous or serrated polyps from the national multicenter project EPIPOLIP and 15 healthy controls with normal colonoscopy. A validated Spanish version of the MEDAS questionnaire was administered to cases and controls.

**Results:**

Adherence to Mediterranean diet was higher in controls than in patients with multiple colonic polyps (MEDAS score: 8.6 ± 1.4 vs. 7.0 ± 1.6; *p* = 0.01). Optimal overall adherence to the Mediterranean diet pattern was significantly higher among the controls than among cases (MEDAS score >9: 46% vs. 13%; OR 0.17; 95% CI 0.03–0.83). Non-optimal adherence to the Mediterranean diet acts as a risk factor for developing colorectal cancer derived from colorectal polyps.

**Conclusion:**

Our results suggest that environmental factors play a role in the pathogenesis of this phenotype.

## 1. Introduction

Colorectal cancer (CRC) is the third most common type of cancer in the world (1). CRC develops from focal changes in benign pre-cancerous colorectal polyps. Patients with multiple colonic polyps of unknown origin are increasingly found in endoscopy units, especially in FIT-based CRC screening programs. The genetic background has been characterized in only 20–30% of cases of multiple colonic polyps (2, 3). The causes of this phenotype are intriguing, and a field effect affecting the colonic mucosa and provoking the development of adenomatous or serrated polyps has been suggested (2). This field effect can be caused by smoking, obesity, metabolic syndrome, unknown genetic alterations, or activation of an inflammatory response. We recently demonstrated an association between activation of a proinflammatory pathway and the development of multiple colonic polyps (2). This route is mediated by the activation of the Th17 immune response that is associated with colorectal tumorigenesis and inflammation-related cancer (4). The etiology of adenomatous and serrated polyps and CRC is multifactorial and varied, and is influenced by modifiable and non-modifiable risk and protective factors (5). Diet is one of the modifiable factors that could be involved in the pathogenesis of colonic polyps (6).

The Mediterranean diet (MD) is the traditional dietary pattern of the Mediterranean region (7). MD is characterized by frequent consumption of foods of plant origin, such as vegetables, fresh fruits, legumes, and whole cereals. Olive oil is the main fat consumed in the MD, together with a moderate consumption of eggs and dairy products, as well as chicken, turkey, and rabbit, and low consumption of red meat and processed products (7, 8). Recently, a positive correlation has been established between the follow-up of a traditional MD and cancer prevention (9, 10). Some studies have shown that dietary patterns based on intake of fruit and vegetables present and anti-tumorigenic effect due to their antioxidants and micronutrients components (11). Moreover, diets with a high consumption of fish can reduce tumor cell growth (12, 13). In addition, the main fat use for cooking in MD is olive oil. This component has polyphenols that also have antioxidant activity and anti-inflammatory effects (10, 14). Regarding CRC, many recent epidemiological studies have shown that adherence to the MD has a protective effect (15–19).

Our aim was to investigate, using a case-control design, the relationship between adherence to the MD and multiple colonic polyps of unknown origin.

## 2. Materials and methods

### 2.1. Participants

**Figure 1:**
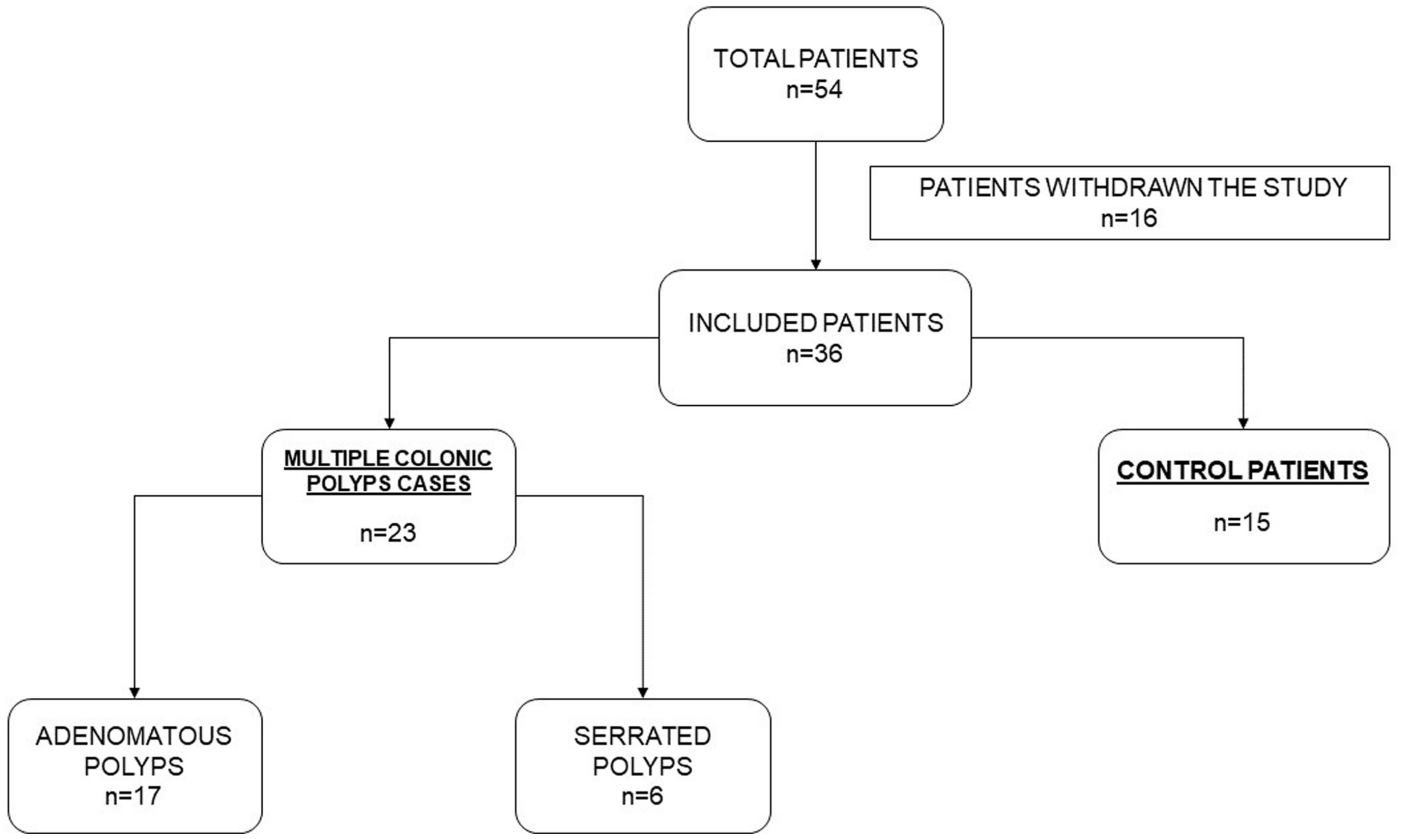
Study flow chart.

The study population consisted of 38 individuals (age: 44–82 years), including 23 cases with multiple colonic polyps from the national multicenter project EPIPOLIP (20) recruited between 2012 and 2014 at the Hospital General Universitario Dr. Balmis in Alicante (Spain) and 15 healthy controls also recruited at the same center among participants in the CRC screening program of the Comunidad Valenciana who had a normal colonoscopy. Patients with multiple colorectal polyps of unknown origin were selected according to the following inclusion criteria: cases with 10 or more polyps (adenomatous or serrated) found in one or different endoscopic examinations. Patients with established causes of polyposis were excluded: polyposis of genetic origin (familial adenomatous polyposis, MUTYH associated polyposis), Lynch syndrome, inflammatory bowel disease. Also, patients with dietary restrictions, such as people with no teeth or following a crushed and restricted diet were excluded. From the original sample of 54 individuals (Figure 1), 16 withdrew for the following reasons: they did not want to participate in the study or were not located after 10 phone calls on different days. In two cases, patients could not be interviewed directly (Alzheimer’s disease in one case and recent throat operation with inability to speak in another), and the interview was conducted with a relative.

Cases and controls were informed about the objective of the study and signed informed consent to participate. The study was approved by the Ethics Committee of the Hospital and followed the principles of the Helsinki Declaration.

### 2.2. Data collection

The interviews were conducted during July 2021 by telephone. The interviews were conducted by a nutritionist (GBM). The collected information included adherence to the MD, physical activity, current and pre-diagnosis smoking, alcohol consumption, presence of diabetes mellitus, consumption of vitamin and/or mineral supplements, and weight and height to calculate body mass index [BMI = weight (in kg)/height^2^ (in m^2^)]. As our population was aged between 44–82 years old, we use World Health Organization criteria for classification of nutritional status for adults over 20 years old (21): Underweight is considered BMI < 18.5 kg/m2; normal weight 18.5 to 24.9 kg/m^2^; pre-obesity 25 and 29.9 kg/m^2^; obesity class 1 between 30 and 34.9 kg/m^2^; obesity class 2 between 35 and 39.9 kg/m^2^ and obesity class <40 kg/m^2^. This classification is also the most universally used, and we believe it is good for purpose of our study. The International Physical Activity Questionnaire (IPAQ) (22) validated for the Spanish population was used to collect information on physical activity, which was classified as high, moderate, or low/inactive. We considered “non-smokers” as those who never smoked or who quit the habit between 2–5 years pre-diagnosis (23, 24). Alcohol consumption information was obtained from open questions (type, amount, frequency) and the variables classified into closed categories based on consumption below or above the established gender-based health risk (25). Indications for colonoscopy (screening or symptoms) were collected from clinical records. Information regarding actual age, age at the moment of diagnosis, gender, and type of polyps (adenomatous or serrated) was also obtained from medical records.

### 2.3. Diet information

Adherence to the MD was assessed using MEDAS, a validated questionnaire for the Spanish population (26). The questionnaire consists of 14 questions with two answers that translate into a score of 1 or 0 depending on the individual’s response (Supplementary Figure S1). The total score can have values between 0 and 14. Optimal adherence was defined as ≥9 points and non-optimal <9 points (27, 28). An additional question regarding alterations in dietary patterns subsequent to diagnosis was incorporated into the questionnaire.

### 2.4. Statistical analysis

Statistical analyses were carried out using SPSS software (version 25). Quantitative variables were presented as mean ± standard deviation (SD) and qualitative variables as absolute frequencies (%). The normal distribution of quantitative variables was verified by the Shapiro–Wilk test and the homogeneity of variances using the Levene test. As all quantitative variables presented a normal distribution, comparisons between groups were performed using the Student *t*-test. The comparisons between groups for qualitative variables were performed using Pearson’s chi-squared test. The multivariate logistic regression model adjusted for age and gender was employed to confirm the significance of variables that show a difference in the univariate model. The odds ratio (OR) for the presence of colorectal polyps was also calculated with 95% confidence intervals (CIs) according to optimal and non-optimal good adherence to the MD score. *p* < 0.05 was considered significant.

## 3. Results

**Figure 2:**
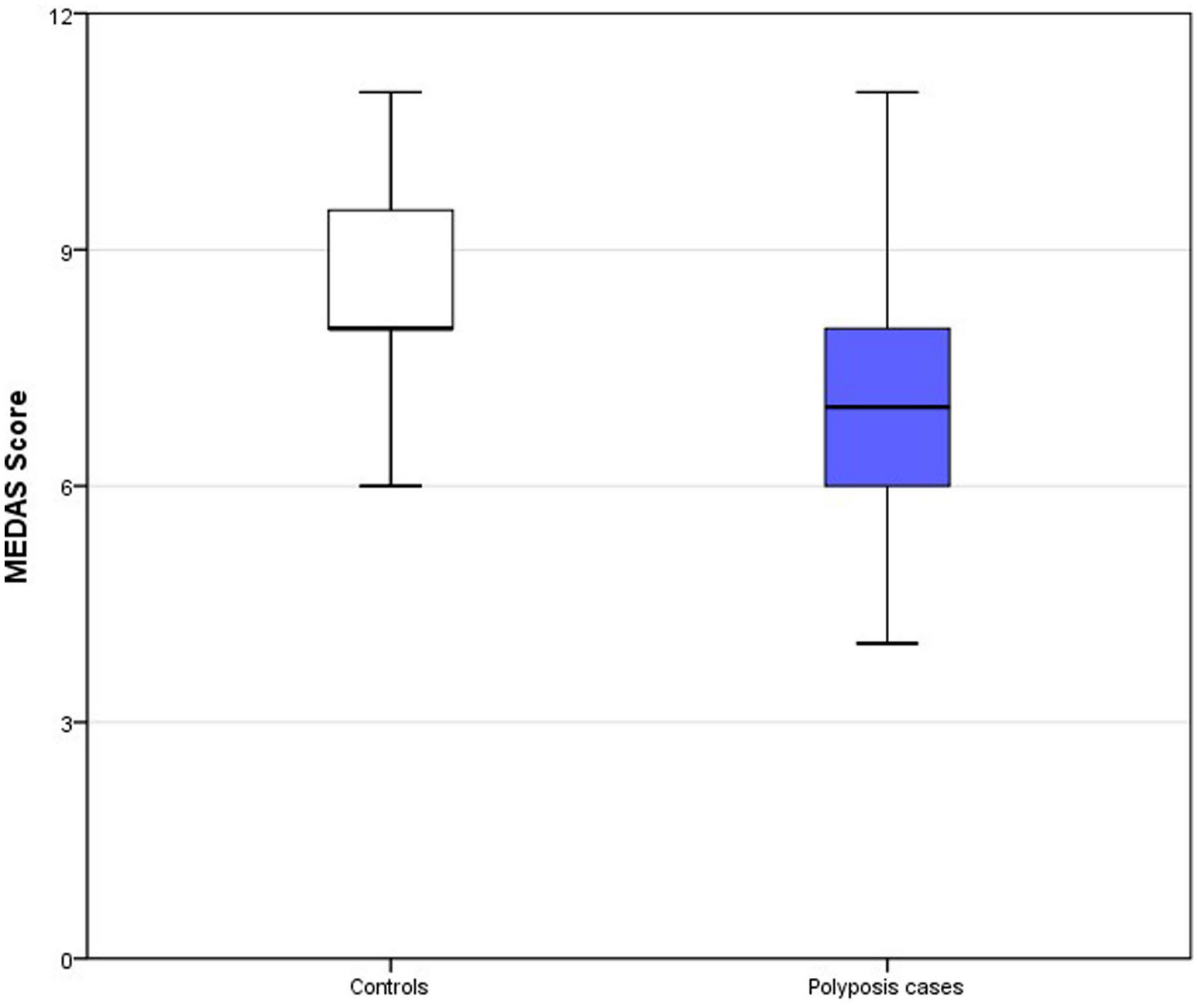
Box-plots MEDAS score. Top of the box represents the 75th percentile (upper quartile), the bottom of the box represents the 25th percentile (lower quartile) and middle represent median. Upper and lower whiskers represent minimum and maximum values.

A total of 38 subjects completed the study: 15 controls and 23 cases of multiple colonic polyps. A total of 17 cases (73.9%) showed a predominance of adenomatous polyps and 6 (26.1%) a predominance of serrated polyps. None of the patients fulfilled criteria for serrated polyposis. Genetic testing for APC and MUTYH was performed in all patients but none of them had a pathogenic mutation in these genes. Clinical and sociodemographic data, as well as adherence to the MD, are given in Table 1. Half of the cases (47.8%) with multiple polyps were considered smokers at the moment of diagnosis versus only 13.3% of controls (*p* = 0.001). Adherence to the MD according to the MEDAS questionnaire score was significantly higher in controls than in patients with polyps (8.6 vs. 7.0, *p* = 0.001) (Table 1 and Figure 2). No significant differences were found for the rest of the variables between cases and controls (age, gender, age at colonoscopy or diagnosis, diabetes mellitus, physical activity level, body mass index, nutritional status, alcohol consumption and supplement consumption). The multivariate analysis including smoking and MEDAS as the only variables significantly associated with multiple colonic polyps in the univariate analysis is shown in Table 2. Only MEDAS score was independently associated with development of the multiple colonic polyps phenotype (OR 1.77; 95% CI 1.05–2.99).

**Table 1:** Sociodemographic and clinical characteristics and adherence to Mediterranean diet.

	Controls (*n* = 15)	Cases (*n* = 23)
Sociodemographic characteristics		
Age (years)	65.7 ± 5.8	66.0 ± 10.0
Gender		
*Men*	6 (40)	15 (65.2)
*Women*	9 (60)	8 (34.8)
Clinical characteristics		
Age at colonoscopy or diagnosis (years)	60.1 ± 5.2	59.8 ± 10.1
Motive for the first colonoscopy		
*Screening*		15 (65.2)
*Symptoms*		8 (34.8)
Type of predominant polyps		
*Adenomatous*		17 (73.9)
*Serrated*		6 (26.1)
Smoker at the moment of colonoscopy or diagnosis		
*Yes*	2 (13.3)	11 (47.8)^a^
*No*	13 (86.7)	12 (52.2)
Diabetes mellitus		
*Yes*	3 (20)	7 (34.4)
*No*	12 (80)	16 (69.6)
Physical activity level		
*Low/inactive*	1 (6.7)	8 (34.8)
*Moderate*	9 (60)	11 (47.8)
*High*	5 (33.3)	4 (17.4)
Body mass index (kg/m^2^)	27.1 ± 3.9	28.0 ± 5.1
Nutritional status		
*Normoweight*	4 (26.7)	5 (21.8)
*Overweight*	6 (40)	12 (52.2)
*Obesity*	5 (33.3)	6 (26.0)
Alcohol consumption		
*Never*	8 (53.3)	4 (17.4)
*Below the risk limit*	6 (40)	17 (73.9)
*Above the risk limit*	1 (6.7)	2 (8.7)
Supplement consumption		
*Yes*	5 (33.3)	8 (34.8)
*No*	10 (66.7)	15 (65.2)
MEDAS (max score = 14)	8.6 ± 1.4	7.0 ± 1.6^b^

Univariate logistic regression analysis. Values are given as *n* (%) or mean ± SD.

a Significant difference (*p* < 0.05) according to Pearson’s chi-squared test.

b Significant difference (*p* < 0.05) according to student *t*-test.

**Table 2:** Multivariate logistic regression adjusted for age and gender with variables associated with the multiple colonic polyps phenotype.

Variable	Odds ratio	95% CI	*p*-value
Age	0.98	0.89–1.08	0.749
Gender	2.81	0.73–10.77	0.116
Smoker at the moment of diagnosis	3.99	0.67–23.83	0.129
MEDAS	1.77	1.05–2.99	0.033

Regarding associations of components of MD and the presence of multiple polyps, controls had an optimal consumption of vegetables per week, as well as a preference for white meat consumption over red meat compared to multiple polyps cases (Table 3). For the rest of the MD components, no significant differences were found between groups. Patients were asked about changes in their diet since the diagnosis of multiple colonic polyps. Twelve of the 23 patients reported no changes in diet, whereas 11 reported changes. All of the reported changes were to improve their diet. Nine patients reported lower intake of processed food, five lower consumption of red meat, and three lower alcohol consumption.

**Table 3:** Components of the Mediterranean diet and presence of multiple colonic polyps.

Diet component	Controls (*n* = 15)	Cases (*n* = 23)
Olive oil as principal source of fat for cooking (Yes = 1 point)	14 (93.3)	22 (95.7)
Use of olive oil (≥4 tablespoons/day = 1 point)^b^	11 (73.3)	13 (54.5)
Vegetable consumption (≥2 servings/day = 1 point)^c^	10 (66.7)	7 (30.4)^a^
Fruit consumption (≥3 pieces/day = 1 point)	7 (46.7)	7 (30.4)
Red meat consumption (<1 serving/day = 1 point)^d^	13 (86.7)	18 (78.3)
Butter/margarine/cream consumption (<1 serving/day = 1 point)^e^	15 (100)	18 (78.8)
Carbonated/sugar-sweetened beverage consumption (<1 cup/day = 1 point)^f^	8 (53.3)	15 (65.2)
Wine consumption (≥7 cups/week = 1 point)^f^	3 (20)	2 (8.7)
Pulse consumption (≥3 servings/week = 1 point)^d^	5 (33.3)	11 (47.8)
Fish/seafood consumption (≥3 servings/week = 1 point)^d^	6 (40)	8 (34.8)
Commercial pastry consumption (<2 servings/week = 1 point)^d^	11 (73.3)	11 (47.8)
Nut consumption (≥3 servings/week = 1 point)^g^	7 (46.7)	7 (30.4)
Preference for white meat consumption over red meat (Yes = 1 point)	15 (100)	17 (73.9)^a^
Consumption of boiled vegetables, pasta, rice, or other dishes with sauce of tomato, garlic, onion, or leeks in olive oil (≥2 times/week = 1 point)	4 (26.7)	6 (26.1)

Values are *n* (%).

a Significant difference (*p* < 0.05) according to Pearson’s chi-squared test.

b 1 tablespoon = 13.5 g.

c 1 serving = 200 g.

d 1 serving = 100–150 g.

e 1 serving = 12 g.

f 1 cup = 100 mL.

g 1 serving = 30 g.

In addition to the differences in the specific items mentioned, the optimal good adherence to the MD score (≥ 9 points) was also significantly higher in the controls than in the cases (Table 4). Similarly, the univariate analysis found that optimal adherence to the MD was inversely associated with multiple colorectal polyps (OR 0.171, 95% CI 0.035–0.834, *p* < 0.021; Table 3). Specifically, an OR of 0.171 estimates a 5.8-times (1/0.171) reduction in the relative risk of suffering from multiple colorectal polyps with optimal adherence to the MD. Conversely, non-optimal adherence to the MD was directly associated with multiple colorectal polyps (OR 5.8, 95% CI 1.200–28.366, *p* < 0.021), resulting in it being a risk factor.

**Table 4:** Estimation of the relative risk of suffering from multiple colorectal polyps according to adherence to the Mediterranean diet pattern.

Good adherence to diet	Controls (*n* = 15)	Cases (*n* = 23)	OR (95% CI)	*p*-value
Optimal	7 (46.7)	3 (13)	0.17 (0.03–0.83)	0.021
Non-optimal	8 (53.3)	20 (87)	5.833 (1.200–28.366)	0.021

Values are given as *n* (%) unless otherwise noted. Optimal adherence was MEDAS score ≥ 9, non-optimal MEDAS score <9. OR, odds ratio; CI, confidence interval.

## 4. Discussion

Our results indicate that adherence to a MD is significantly higher in controls than in cases with multiple colorectal polyps. When differentiating between optimal and non-optimal adherence to the MD (score ≥9 vs. <9), non-optimal adherence was a risk factor for the development of the disease. The results are consistent with other studies and meta-analyses reporting inverse associations between adherence to a MD and the incidence of colorectal polyps (29–34), but this is the first study showing an association between diet and this specific phenotype of multiple colonic polyps. These findings emphasize the potential role of environmental factors, such as diet and lifestyle, on the development of the phenotype of multiple colonic polyps of unknown origin, adding one more piece to the puzzle of this intriguing condition.

There are several potential pathophysiological explanations for the association between adherence to a MD and the development of multiple colonic polyps. For example, some of the components of the MD, such as olive oil, legumes, fresh fruits, nuts, vegetables, and fish, contain antioxidant and anti-inflammatory compounds that may contribute to minimizing cell degeneration and the proliferation of cancer cells (9, 10). Adherence to the MD improves the oxidative and inflammatory status by reducing the level of different circulating cytokines (35). Polyphenols in the MD are able to modify gut microbiota, promoting antioxidant effects (36). In addition, resveratrol has been linked to apoptosis, cell cycle regulation, and epithelial-mesenchymal transition (37, 38). Adherence to a MD affects the microbiome and can modify different aspects of the intestinal immune response. Some of these aspects, such as activation of the Th17 response with increased production of IL-17/IL-23 cytokines, have also been related to the development of multiple colonic polyps of unknown origin (2).

Several studies have established correlations between a MD and incidence of colorectal polyps. In this context, a population-based case-control study of 783 individuals found significant inverse associations between the incidence of advanced colorectal polyps and high adherence to a MD with high fish consumption, low consumption of sugar drinks, and low consumption of red meat (34). An additional case-control study demonstrated that higher adherence to a MD was significantly associated with a lower risk of adenomatous polyps, as well as CRC (29). Two other hospital studies found that higher levels of adherence to a MD were significantly associated with a lower risk of developing distal colorectal adenomatous polyps in men (30, 31). Another European study of women with no prior history of colorectal adenomas, but diagnosed with adenomatous polyps on an initial colonoscopy, found that following a MD for 3 years after diagnosis offered protective effects against polyp recurrence (33). Finally, a study conducted in the United States analyzed associations between diet and the risk of colorectal polyps in 2818 subjects, finding significant associations between a lower risk of colorectal polyps and high consumption of green vegetables, dried fruits, legumes, and brown rice (39).

A more detailed analysis of diet components showed that controls reported preferential consumption of white meat over red meat compared to multiple colonic polyps cases. This observation is consistent with similar studies and systematic reviews (9, 31, 34, 40). It is important to separately study the consumption of white and red meat, due to the different roles that both can play in the carcinogenic process. Red meats contain higher concentrations of heme iron than white meats which leads to endogenous formation of carcinogenic N-nitroso compounds (NOCs) and lipid peroxidation that contribute to the development of CRC. In addition, white meats are richer in polyunsaturated fatty acids (PUFAs). PUFAs inhibit the synthesis of proinflammatory cytokines and are considered to reduce carcinogenesis by inducing apoptosis and controlling cell cycle and eicosanoid production (41, 42). Red meat and its association with colorectal polyps and CRC has been observed in several studies (43–45). Red meat is classified by the IARC as “probably carcinogenic” to humans. In addition, vegetable consumption was significantly higher in controls than in cases with multiple polyps, which also agrees with previous studies (46, 47). For example, two studies, one cohort and one case-control, found that the risk of colorectal polyps is increased with lower consumption of cooked green vegetables (39) and fruits and vegetables (40), respectively. Another retrospective case-control study found that vegetable consumption is inversely associated with CRC development (48). A systematic review of adherence to a MD and cancer showed that the relative risk of cancer increases inversely with vegetable consumption (9). However, no differences were found in fruit consumption between polyposis cases and controls, which is in contrast to previous studies (34, 40). These studies have shown that fruit consumption is a protective factor for both polyp formation and CRC. A possible explanation could be related to the usually high consumption of fruits in the Spanish population.

Nutritional and dietary education is a critical component for healthcare professionals in delivering comprehensive care to patients with digestive disorders. Through the provision of evidence-based guidance on balanced diets and adopting a healthy lifestyle, healthcare professionals can effectively promote optimal intestinal health while mitigating the risk of inflammation-associated diseases. Simultaneously, it is imperative to caution against the consumption of foods known to trigger inflammatory conditions. The implementation of effective nutritional education strategies not only contributes to the management of digestive disorders but also enhances patients’ overall quality of life, fostering optimal intestinal health outcomes.

This study has several limitations. First, it is not possible to evaluate the pre-diagnostic diet in real time, taking into account that the studied population was not recently diagnosed. We attempted to correct for this limitation by asking cases if they made any significant changes to their diet since diagnosis. Among those who made changes, the modifications improved diet quality. All of the changes were located in some items of MEDAS. However, according to the qualitative information obtained, the pre-diagnosis MEDAS score would be lower than the current score obtained in the present study. Nevertheless, the adherence to the MD by these patients was still low. The second limitation refers to the use of the validated MEDAS questionnaire with no possibility for modifications. In this context, it was not possible to make more detailed discriminations in some dietary parameters, such as the consumption of red meat. MEDAS only differentiates between a preferential consumption of red vs. white meat, making it difficult to assess the servings of each type of meat. Another limitation is the small sample size, which makes it difficult to associate between components of the MD specifically related to development of the multiple polyps phenotype. Moreover, the population of patients with multiple polyps was not homogeneous, indistinctly including patients with multiple adenomas and serrated polyps.

## 5. Conclusion

The findings of this study indicate that optimal adherence to a MD assessed using the validated MEDAS questionnaire is inversely associated with the risk of multiple colorectal polyps. The results serve as theoretical support for a potential intervention at the primary care level aiming to prevent colorectal polyposis through the promotion of a traditional MD and incorporation of this diet into current lifestyles. However, these results must be validated in a prospective study with a larger sample of patients with multiple polyps.

## Data availability statement

The raw data supporting the conclusions of this article will be made available by the authors, without undue reservation.

## Ethics statement

The studies involving human participants were reviewed and approved by Comite Etico del Hospital General Universitario Dr. Blamis. The patients/participants provided their written informed consent to participate in this study.

## Author contributions

GB-M, AM-R, AG-H, ER, and RJ: conceptualization. GB-M, AM-R, AG-H, DG-B, ER, and RJ: investigation, writing—article and editing, and supervision. GB-M, AM-R, AG-H, ER, and RJ: writing—original draft preparation. GB-M and RJ: funding acquisition. All authors contributed to the article and approved the submitted version.

## Funding

This work was supported by a grant from the AECC Scientific Foundation “Ayuda Programa Prácticas Laboratorio Verano AECC 2021” (PPLAB211883BUJA) and Instituto de Salud Carlos III (PI08/0726 and PI20/01527). AM-R received a pre-doctoral grant from Instituto de Salud Carlos III (FI18/00301). AG-H received a Sara Borrell grant from the Instituto de Salud Carlos III (CD19/00133). Asociación para la Investigación en Gastroenterología de la Provincia de Alicante (AIGPA), a private association that promotes research in gastrointestinal diseases in Alicante.

## Conflict of interest

The authors declare that the research was conducted in the absence of any commercial or financial relationships that could be construed as a potential conflict of interest.

## Publisher’s note

All claims expressed in this article are solely those of the authors and do not necessarily represent those of their affiliated organizations, or those of the publisher, the editors and the reviewers. Any product that may be evaluated in this article, or claim that may be made by its manufacturer, is not guaranteed or endorsed by the publisher.
