# Supplementary material for: Adherence to Mediterranean diet and its association with multiple colonic polyps of unknown origin: a case-control study
Source: Front Nutr. 2023 Jun 22;10:1186808. doi: 10.3389/fnut.2023.1186808 (PMC10324649; doi:10.3389/fnut.2023.1186808)
Supplement: Supplementary file 1 [file Data_Sheet_1.docx]

Supplementary Material

**Adherence to Mediterranean diet and its association with multiple colonic polyps of unknown origin: a case-control study**

Gabriela Bujanda-Miguel^1^†, Alejandro Martinez-Roca^1^†, Anabel García-Heredia^1^, David Guill-Berbegal^1^, Enrique Roche^1,2,3^, Rodrigo Jover^1,4*^

^1^ Servicio de Medicina Digestiva. Hospital General Universitario Dr. Balmis. Instituto de Investigación Biomédica ISABIAL, 03010-Alicante, Spain.

^2^ Department of Applied Biology-Nutrition, Institute of Bioengineering, University Miguel Hernández, 03202-Elche, Spain.

^3^ CIBER Fisiopatología de la Obesidad y Nutrición (CIBEROBN), Instituto de Salud Carlos III (ISCIII), 28029-Madrid, Spain.

^4^ Departamento de Medicina Clínica, Universidad Miguel Hernández, Alicante, Spain.

†**Equal contribution and first authorship:**

Gabriela Bujanda-Miguel†, Alejandro Martinez-Roca†**.** These authors contributed equally to this work and share first authorship.

*** Correspondence:**Rodrigo Jover; [rodrigojover@gmail.com](mailto:rodrigojover@gmail.com)

Enrique Roche; [eroche@umh.es](mailto:eroche@umh.es)

## Supplementary Figures


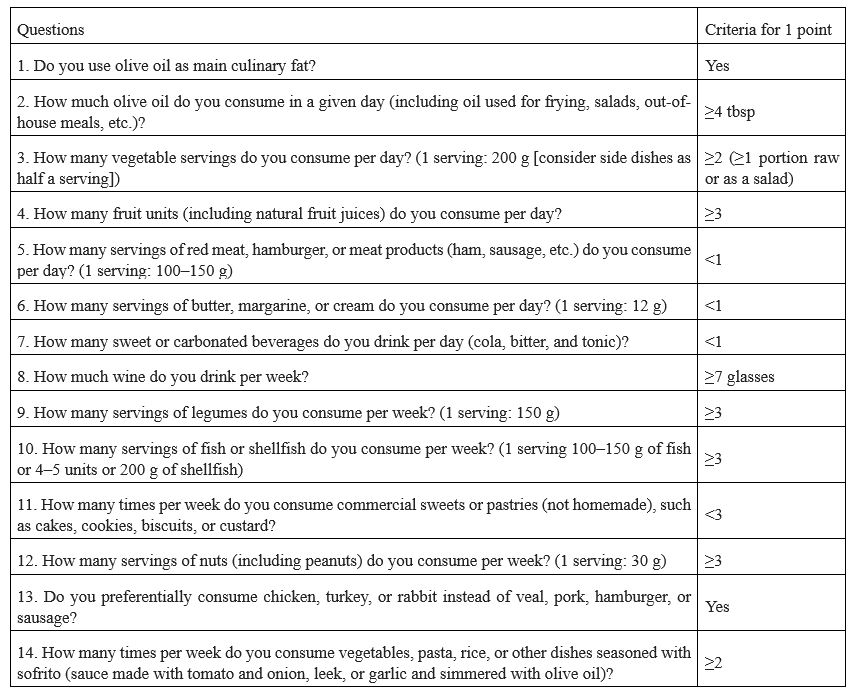


**Supplementary figure 1.** MEDAS-14 questionnaire. Adapted from Spanish version.
